# Perspective in infertility: the ovarian stem cells

**DOI:** 10.1186/s13048-015-0184-9

**Published:** 2015-08-07

**Authors:** Erica Silvestris, Stella D’Oronzo, Paola Cafforio, Giuseppe D’Amato, Giuseppe Loverro

**Affiliations:** Department of Biomedical Sciences and Human Oncology, Division of Gynecology and Obstetrics, University of Bari ‘Aldo Moro’, P.za Giulio Cesare, 11, Bari, 70124 Italy; Department of Biomedical Sciences and Human Oncology, Division of Molecular Oncology, University of Bari ‘Aldo Moro’, Conversano, Bari Italy; Department of Biomedical Sciences and Human Oncology, ASL Bari, PT Jaia, Reproduction and IVF Unit, University of Bari ‘Aldo Moro’, Conversano, Bari Italy

## Abstract

Infertility is a medical and social condition that affects millions of women worldwide and is today considered so far as a new disease. A considerable progress has been recently pursued in the field of the reproductive medicine and the infertility treatment may account for novel and modern procedures such as *in vitro* oocyte fertilization, egg donation, pregnancy surrogacy and preimplantation diagnosis. However, great interest has lately been reserved to the ovarian stem cells (OSCs) whose existence in woman ovaries has been proven. OSCs are thus suitable for developmental studies in infertility and in other clinical applications as endocrine derangements due to premature ovarian failure, or for infertility treatment after cancer chemotherapies, as well as in restoring the hormonal balance in postmenopausal age.

## Introduction

Infertility is a prevalent problem in our society today. The WHO estimates its worldwide diffusion near to 80 millions of women with probably the highest incidence in developing countries for the absence of health programs to prevent infectious and sexually transmitted diseases in young women. On the other hand, in western countries the infertile female population is increasing, and is apparently related to multiple endocrine dysfunctions also associated to correlated lifestyle habits. For instance, psycodyslettic drugs, as well as caffeine, alcohol abuse and tobacco, variably interfere with the hepatic metabolism of estrogens whose defective bioavailability primarily concurs to cause infertility in association to other conditions as the work-related stress, anxiety, obesity dependent on hypercaloric food assumption, or vice versa, chronic utilization of weight-losing diets at low protein content. Notably, the defective energy metabolism for extreme diets during agonistic sport activities may cause the ‘*female athlete triad*’ a new syndrome characterized by eating disorders, osteoporosis, and oligomenorrea leading to amenorrea and sterility [1].

During the past two decades, modern infertility treatments as *in vitro* oocytes fertilization, egg donation, pregnancy surrogacy, heterologous artificial fertilization and preimplantation diagnosis (PGD), have reduced of approximately 10 % the diffusion of this disease. Furthermore, in parallel with the increasing adoption of the heterologous fertilization, novel technologies as the genomic gamete screening by gene sequencing analysis, are currently used to avoid pregnancies at risk for genetically inherited diseases. Moreover, infertility treatments also take advantage from innovative ovotechnology methodologies and a large interest by both basic science and clinical investigators is today reserved to the ovarian stem cells (OSC) whose demonstration in human ovaries is at present still debated.

Beyond the multifactorial pathogenesis of infertility, in this review we will briefly comment the major approaches for the treatment of infertility that are world-wide adopted and will revisit the literature supporting the existence of the OSCs in the woman ovaries in parallel with their specific molecular characters.

### Current approaches for woman infertility care

The approaches for treating the woman infertility include the functional organ recovering and the substitutive treatments programmed in relation to the disease pathogenesis as summarized in Table 1.

**Table 1:** Schematic list of major procedures for treatment of infertility

	Indication	Success rate	Advantages	Disadvantages
IUI	- Unexplained infertility	variable: ~15-20 %	- Very simple procedure	Virtually no disadvantages
		Factors reducing the success rate:		
			- High concentration of motile sperms	
		- Older age of the woman		
	- Mild/moderate male infertility			
		- Poor egg quality		
		- Poor sperm quality		
	- Cervical factor infertility	- Severe endometriosis		
		- Severe damage to fallopian tubes		
		- Blockage of fallopian tubes		
	- Minimal endometriosis			
IVF	- Unexplained infertility	~30 % of all treatment cycles (depending on the age of the woman)	- Treatment of choice with donor eggs	- Multiple pregnancies
	- Low sperm numbers or motility			
			- Reduce surgery on damages tubes	
	- Infrequent or absent ovulation			
	- Tubal factor infertility			
	- Cervical factor infertility			
	- Immunological factor infertility			
				- OHSS.
				- Ectopic pregnancy
	- Moderate or severe endometriosis			- Painful treatment
				- Expensive treatment
	- Age-related infertility			
	- Failure of other fertility treatments			
ICSI	- Low numbers of motile sperm	variable: ~8.5 % to 33.5 % (age range between 35 and 44)	- Overcomes male infertility	- Higher risk of miscarriage
	- Abnormal morphology sperm			
			- Efficient where few eggs are available	
	- High levels of antisperm antibodies			
				- Success depends on different factors (age of female, quality of eggs, sperm, uterus)
	- Prior or repeated fertilization failure			
	- Limited frozen sperm			
	- Obstruction of the male tract sperm			
				- Painful treatment
				- Expensive treatment

*IUI* Intrauterine insemination, *IVF* In vitro fertilization, *ICSI* Intracyplasmatic sperm injection

#### Recovering the ovary function

Both hypogonadotropism or hypergonadotropism concur to ovary failure in approximately 20 % of the infertile women and the clomiphene citrate (CC) has been widely adopted in the past for generic ovulatory dysfunctions. Recently, the metformin has been shown to improve the rate of ovulation in particular in the polycystic ovary syndrome unresponsive to CC and/or to gonadotropins [2].

#### Recovering the uterus function

The intrauterine semen insemination is recommended for the infertility due to cervical factors or to the male ejaculatory dysfunction. The procedure is simple since the donor sperm is inseminated in parallel with induction of the ovulation, but the success rate is not so high.

#### *In vitro* fertilization (IVF)

This procedure was initially developed in 1978 by Steptoe and co-workers for treating the tubal dependent infertility but is currently used for other types of infertility [3]. The procedure includes four sequential steps as follows:

- *ovulation induction -* The success rate is directly related to induction of ovulation and then to the number of oocytes retrieved and embryos available for the transfer.
- *oocyte insemination -* This procedure is based on the recovery of ovarian follicles by ultrasonography and subsequent selection of the suitable eggs to be cultured in the presence of the sperm sample. The intracytoplasmic sperm insemination is preferred in the presence of failure of the IVF.
- *embryo transfer -* The embryo transfer is usually performed 72 hours after the egg retrieval. In relation to the woman’s age and previous pregnancy history, two to three embryos are transferred into the uterine cavity under ultrasound guidance by an echogenic catheter placed into the uterine cavity through the cervical canal. Since the ovulatory cycles take advantage from progesterone supplement, during the day after the egg retrieval, to support the luteal phase it is frequently administered progesterone either intra-vaginally or intramuscularly [4].

### Perspective: the ovarian stem cells

Although the stemness studies have greatly improved the basic knowledge of hematopoietic and mesenchymal stem cells (MSC) in humans providing substantial progress in different cytotherapies as bone marrow (BM) transplantation or anti-cancer approaches with engineered MSCs, poor information is presently available on the biology of OSC whose existence in woman ovaries is today still disputed although proven.

#### Historical background

Preliminary observation on OSCs dates on 1951 by Zuckerman et al. [5]. They asserted a long-held dogma according to which in postnatal mammalian ovaries of most species no renewable germinal OSCs are available and, thus, supporting the hypothesis that during the lifetime a numerically fixed pool of oocytes are committed for the fertility. This original pool would account in woman for approximately 10^6^ oocytes in puberty, but the number declines with aging until exhaustion at menopause.

This assumption was subsequently refuted by Tilly and co-workers [6], who postulated the presence of mitotically active OSCs in both juvenile and adult murine ovaries capable to warranty both oocyte and follicle availability after birth. In their experiments, they observed a discordance between the rate of follicle depletion and the reproductive lifespan and found normal histological aspect in ovaries from mice receiving Busulfan showing healthy maturing follicles and corpora lutea. Thus, they concluded that oocytes underwent normal differentiation in these ovaries and grafted ovarian fragments from adult wild type mice in ovaries of green fluorescent protein (GFP)-expressing mice with the aim to explore the occurrence of GFP-positive oocytes in wild-type grafts. The granulosa cells surrounding GFP-positive oocytes within the transplanted fragment were found to be GFP-negative thus suggesting that transgenic OSCs migrated into the graft and formed new follicles in adult mice. Immediate criticisms were raised to these observations and focused not only the methods used to distinguish healthy from unhealthy follicles, but also the computation system used as well as the Busulfan treatment which is a well-known cytotoxic agent for spermatogonial cells, but with unknown effects on OSCs [5].

Other investigators confuted the Tilly’s hypothesis and simulated the dynamics of murine follicle progression during the mouse lifespan by employing two mathematical models, namely the *‘stem cell’* and the *‘fixed pool’* model and argued that the physiological decline in follicle populations supported the theory of *‘fixed pool population’* of oocytes as lifetime ovarian reserve [7]. However, the debate continued when Tilly and colleagues speculated the existence of a putative reservoir of OSCs in the BM of adult mice. In fact, they primarily described the expression of germ cell line markers including Oct4, MVH (*mouse vasa homologue*), DazI, Stella and Fragilis in BM from adult female mice and then transplanted their BM into adult females pre-sterilized with both Cyclophosphamide and Busulfan, and detected a significant generation of new oocyte-containing follicles and corpora lutea [8]. This result was confirmed a few years later by transplanting BM from GFP-expressing into wild type mice previously sterilized with Cyclophosphamide and Busulfan. The offspring produced by treated females derived from the recipient germline compartment, suggested that the transplantation rescued fertility by either protecting existing oocytes from cytotoxic agents, or reestablishing in the host the somatic cell population, necessary to reinstitute oogenesis [9]. Thus, these preliminary observations gave rise to further studies aimed at OSC isolation and transfer into sterile animals to recover their fertility.

#### Detection and isolation of OSCs

The first attempt to isolate and culture germinal OSCs in mammals was completed by Zou and co-workers who purified neonatal and adult female OSCs from mice through a cell-sorting approach [10]. They first performed a two-step enzymatic digestion of murine ovarian tissue followed by immunomagnetic isolation of cells expressing the germinal marker MVH, also known as Ddx-4 (*DEAD box polypeptide 4*). This protein is a 79.3 kDa peptide with the extracellular COOH-domain and a cytoplasmatic tale that is uniquely expressed by germinal ovarian and spermatogonial cells [11]. After culturing OSCs from neonatal and adult mice for more than 15 and 6 months respectively, the Authors infected the isolated Ddx-4^+^ cells with the MSCV-PGK-GFP virus and transplanted them into ovaries of infertile female mice. They ultimately observed that the animals produced GFP^+^/Ddx-4^+^ oocytes.

**Fig. 1:**
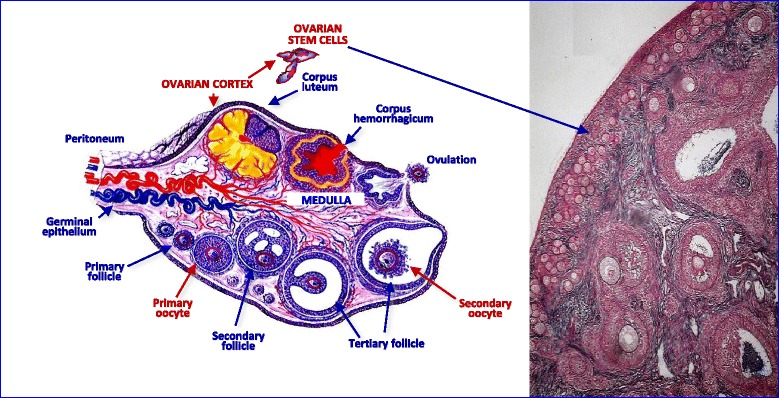
Structure of human ovary. *Left* – Schematic representation of the ovarian structure in a woman in reproductive age, showing the evolution of primary follicles to corpora lutea that cyclically occur in the cortex of the organ. *Right* – The ovarian cortex is the presumable site of the ovarian stem cell location in this ovary preparation after hematoxylin/eosin staining

Therefore, Tilly and collaborators validated a FACS-based protocol to purify OSCs from both murine and human ovarian cortex (Fig. 1) as putative location [12]. The investigation was based on the immunological detection of a putative cell surface variant of Ddx-4, commonly considered cytoplasmic, through a rabbit polyclonal antibody against the COOH-terminus of the protein. Once purified by sorting, the cells showed a germline gene expression pattern and were thus established in culture, engineered to express GFP and injected into biopsies of adult human ovarian cortical tissue. The fragments were subsequently xenografted into immunodeficient female mice and new follicles containing GFP-positive oocytes were detected 1–2 weeks later. Major skepticism arose with respect to the choice of using Ddx-4 as cell surface marker used for the OSC sorting since its localization apparently occurs in cytoplasm rather than on plasma membrane [13]. On the other hand, to strengthen the criticism, Zhang and colleagues [14] generated a fluorescent mouse model for both in vivo and in vitro tracing of Ddx-4 expressing OSCs, and in the absence of fluorescence in ovaries they stated that OSCs did not enter in mitosis, nor contributed to the oocyte renewal. Table 2 lists an incomplete panel of markers differentially expressed by OSCs and oocytes.

**Table 2:** Molecular markers differentially expressed by OSCs and oocytes. Only few markers are maintained in the differentiating process. The cell membrane isoforms of Ddx-4 and SSEA-4 are detectable on OSCs [12, 15, 17, 23–26]

Ovarian stem cells	Oocytes
Ddx-4 (cell membrane and cytoplasm)	Ddx-4 (cytoplasm)
SSEA-4 (cell membrane and cytoplasm)	SSEA-4 (cytoplasm)
OCT-4 A and B	OCT-4 B
c-kit	c-kit
DAZL (nucleus and cytoplasm)	DAZL (cytoplasm)
Fragilis	ZP (zona pellucida proteins)
CD133	GDF-9 (growth differentiation factor 9)
Stella	NOBOX (newborn ovary hemeobox protein)
Nanog	SCP-3 (synaptonemal complex protein 3)
Sox-2	
Blimp-1	

Another group of investigators described the presence of both very small embryonic-like stem cells (VSELs) and germinal OSCs within the ovarian surface epithelium (OSE) of adult mammals. To obtain these cells from OSE, the samples were enzymatically digested or mechanically scraped and the putative OSCs were analyzed for the expression of OCT-4A, SSEA-4(*stage specific embryonic antigen-4*), Fragilis, CD133 and others. These cells were then maintained in continuing cultures for 21 days and underwent spontaneous differentiation into oocyte-like structures suggesting that to identify OSCs from ovaries it is preferable to use stemness markers rather than Ddx-4 [15]. Based on these observations, the same Authors set up an immunomagnetic sorting of OSE stem cells from sheep ovaries using SSEA-4 as major surface marker. Therefore, the cells were typed by immunocytochemistry for specific markers of both staminal and germ line profiles as well as by immunofluorescence and RT-PCR, and then cultured for 3 weeks. The SSEA-4^+^ OSE stem cells from those animals underwent dynamic changes resembling spontaneous differentiation into oocyte-like structures [16].

In subsequent studies, Zou and co-workers [17] further tried to isolate OSCs by magnetic sorting through three germinal markers, namely CD9, Stpb-c (*short-type pituitary gland and brain-cadherin*) and Fragilis. However, the isolation of OSCs efficiently turned out only by targeting the protein Fragilis, since CD9 was detected on membranes of both germ and somatic cells, including those of granulosa, whereas Stpb-c was inapplicable since the appropriate primary antibody was actually unavailable.

**Fig. 2:**
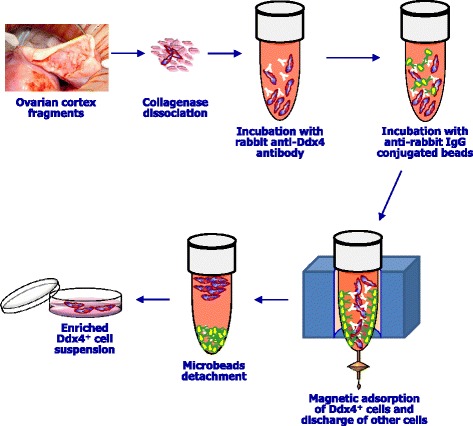
Isolation of OSCs. Representative procedure of OSC separation from ovarian cortex, incubation with rabbit anti-Ddx4 antibody, subsequent incubation with anti-rabbit IgG conjugated with magnetic beads, capture of cells by magnet and further separation through MACS column leading to collection of Ddx4^+^ cells, detachment form the beads and elution in medium to obtain enriched OSC preparations

Based on these different procedures, in an attempt to investigate the occurrence of OSCs in ovaries from adult women, we tried to isolate these cells from freshly collected ovarian cortical tissue, using a Ddx-4 antibody-based magnetic sorting, as described by Woods and Tilly [18]. Fig. 2 depicts the major steps of this procedure.

**Fig. 3:**
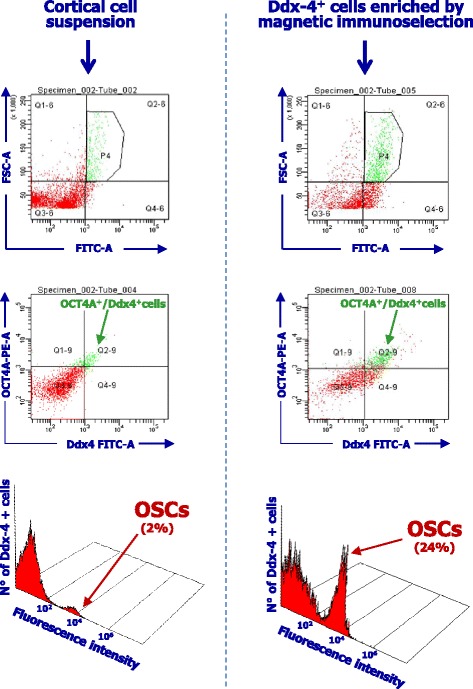
Flow cytometry analysis of OSCs. Fluorescence flow cytometry analysis of OSCs before (left) and after (right) the separation by the immunomagnetic procedure. The population was first gated (P4) in relation to the cell size and then assessed in double fluorescence analysis to detect Ddx4 on ovarian cells expressing OCT4A antigen as ontogenetic marker. As shown, the OCT4A^+^/Ddx4^+^ cell population was expanded after the immunomagnetic selection (middle), since the Ddx4^+^ cell population was increased up to 24 % (right) of the original 2 % (left) value in the initial cortical ovarian suspension, with a remarkable fluorescence intensity suggesting the high molecular expression of Ddx4 molecule

**Fig. 4:**
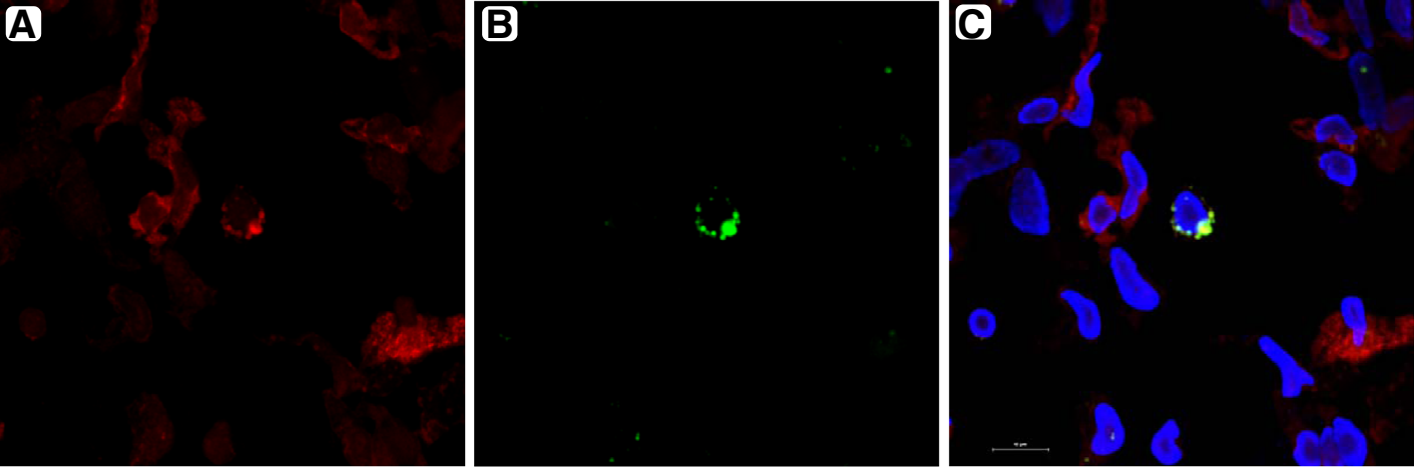
Ddx4 localization on immunomagnetic isolated OSC. Confocal microscopy showing a single OSC of small size with large nuclei counterstained by DAPI (C) and few cytoplasm. Ddx4, revealed by FITC (green) in B, localizes in vicinity of the membrane actin (red in A). By merging both FITC and PE, Ddx4 is localized outside of the nucleus and shows high fluorescence intensity. Other nuclei stained by DAPI belong to Ddx4-negative cells within the preparation

To verify the enrichment in OSCs of the cell suspension obtained by immunomagnetic selection, we evaluated their phenotype by flow cytometry using both FITC-conjugated anti-Ddx-4 and PE-conjugated anti-human OCT4A as ovary lineage specific marker. The flow cytometry analysis is illustrated in Fig. 3. The Ddx4^+^ cell population was increased up to 24 % (lower section: right) of the original 2 % (left) value as detected in the initial cortical ovarian suspension, with a remarkable fluorescence intensity suggesting the high expression of Ddx4 molecule. Ddx4 was also detected by confocal microscopy revealing high Ddx4 fluorescence intensity that supported the flow cytometry data (Fig. 4). Therefore, the evidence of Ddx4 on OCT4A^+^ cells from ovarian cortex by both flow cytometry and fluorescence microscopy provided further evidence to the existence of OSCs in the human ovary and that their sorting is reliable by appropriate combined separation methodology.

#### Concurrent research on human OSCs

Once isolated from the OSE, OSCs have been investigated from several Authors for their potential application in infertility treatment. Indeed, Hayashi and coworkers induced murine female embryonic stem cells to differentiate into primordial germ cell–like cells (PGCLCs) that were utilized to reconstitute ovaries. After transplantation under the ovarian bursa of adult mice, the reconstituted ovaries were then isolated and histologically analyzed, and the PGCLCs were revealed to contribute to the oocyte formation. Once reaching metaphase II, these cells were thus fertilized and generated two-cell embryos that, transferred into foster mothers, developed pups able to reach adulthood [19].

If working on animal models was quite easy, direct investigation of OSCs in humans revealed more troubles both for ethical issues and for the scarcity of OSCs in adult OSE. Among the groups exploring this topic, Stimpfel and co-workers successfully characterized and differentiated in vitro stem cells from the adult human ovarian cortex [20]. The isolated cells expressed pluripotency markers as alkaline phosphatase, SSEA-4 and OCT4 together with Ddx4 as prototypic ovarian germ line marker. These cells exhibited a high degree of plasticity since they, once adequately stimulated, differentiated in various somatic cells of mesoderm, ectoderm and endoderm layers, without forming teratomas in immunodeficient mice. However, this may also depend on the cell exiguity as well as on their slow proliferation.

At present, an intriguing theory postulates that the impaired bioenergetics capacity in oocytes correlates with the decline of quality in both eggs and embryos in particular in advanced age of fertile women since recurrent morphologic alterations are frequently observed in the oocyte mitochondria of adult women. This concern led to define a clinical protocol using the oocyte cytoplasm extracted from a young donor that was transferred into the eggs of the recipient woman. The procedure, however, had short life because of concerns regarding the consequent mithocondrial heteroplasmy, whose result was that children generated by this protocol possessed three different sources of genetic material, namely the biological parents and the oocyte donor, for the presence of maternally inherited DNA in mithocondria. Therefore, according to the U.S. Food and Drug Administration (FDA), this procedure was considered a genetic manipulation of human germ cells and was no longer applied [21].

The discovery that OSCs are present in fertile women ovaries has driven to an emerging modified version of ooplasmic transfer, called AUGMENT (*autologous germline mitochondrial energy transfer*) in which autologous germline-derived cytoplasmic extract or purified mitochondria can be used for the bioenergetics reinforcement of oocytes. Another potential approach to overcome the defective egg energy includes the improvement of both biological and chemical compounds bioavailability, with the purpose to enrich the mitochondrial number or the efficacy of ATP production in oocytes [21, 22].

These studies emphasize the increasing interest in human OSCs for future application in treatment of human infertility that undoubtedly needs further investigation in this specific topic.

### OSCs in prospective applications

As described, the female infertility occurs as dependent on different pathologies producing the pre-mature depletion of the woman oocyte pool due to a number of disorders as autoimmune diseases, X-chromosome linked genetic disorders, environmental hazards, oophorectomy for benign or malignant ovarian neoplasms, as juvenile endometriosis or ovarian cysts, and premenopausal ovarian cancers respectively. Furthermore, the conventional chemotherapy treatments are the most common causes of infertility in female cancer patients.

Highly recurrent cancers in preadolescent and reproductive age include leukemia, lymphomas and other hematologic malignancies, together with breast and cervical cancers, sarcomas, brain and kidney neoplasms. Since cytotoxic treatments primarily exert anti-mitotic effect to both disable the cell division and inhibit the DNA replication, ovarian exposition to these drugs leads to irreversible follicular and oocyte damage, resulting in infertility. However, several techniques as the oocyte and ovarian tissue cryopreservation followed by autologous oocyte transplantation, embryo freezing, and, recently, *in vitro* culture of ovarian tissue, follicles and oocytes are currently used to prevent the damage of nuclear content and preserve from iatrogenic sterility. While most of these techniques are considered for future applications, the embryo freezing is a method commonly used for fertility preservation in the majority of women in cancer treatment.

These techniques are not entirely safe since, as effect of the hormone stimulation for the maturation of the oocyte pool, estrogen-sensitive tumors as both breast and endometrium cancers may rapidly grow before the chemotherapy treatment. On the other hand, the techniques based on cryopreservation of ovarian tissue after hormonal stimulation and subsequent autologous transplantation, imply the risk of reintroducing malignant cells, especially in patients with leukemia who may harbor malignant cells in their bloodstream [13].

Although some Authors persist in assuming that primordial follicles are sufficient to sustain adult oogenesis both in mice and humans, without requiring OSCs, isolation, establishment in culture and propagation of these cells would open the door to a novel fertility preservation approach, especially in women with iatrogenic premature ovarian failure [23–26]. In addition, such a novel therapeutic approach would be useful for women suffering of common post-menopausal health complications related to hormonal imbalance, as cardiovascular diseases, osteoporosis, cognitive decline and depression [27].

## Conclusion

Infertility is both a medical and a social problem that affects a large female population worldwide and is undoubtedly a disease. It is associated to a number of pathophysiological conditions and its pathogenesis is frequently undefined, with relative uncertainty to establish the appropriate treatment choices. If in developing countries the major reason of this disease is related to the scarcity of medical care and to defective information, its increasing incidence in industrialized countries may also depend on ovarian endocrine dysfunctions related to the life-styles.

In the last decades, a number of treatments have been introduced for this disease and the field of reproductive medicine is presently in rapid evolution for the utilization of novel therapeutic approaches, primarily based on the in vitro fertilization. However, new perspectives in infertility treatment derive from the studies on OSCs originally pursued by Tilly and co-workers. Based on preliminary observations in mice, they postulated the presence of mitotically active OSCs in human ovarian cortex, capable to ensure oocyte turnover during the women lifespan. Despite initial skepticism, OSCs have been convincingly detected in the woman, thus opening a new chapter in reproductive medicine. The future applications of these cells to infertility field will cover different and unrelated conditions ranging from the endocrine defective ovarian reserve to the iatrogenic infertility in oncologic patients, as well as the utilization of OSCs only for restore the woman endocrine physiology in postmenopausal age.

Based on the availability of novel and potent technologies in the field of the reproductive medicine, it is desirable that these emerging evidences give a contribution to the solution of the problems related to infertility, in particular in developing countries, where this disease is largely diffused.
